# Anti-tumor effects of rigosertib in high-risk neuroblastoma

**DOI:** 10.1016/j.tranon.2021.101149

**Published:** 2021-06-09

**Authors:** Katarzyna Radke, Karin Hansson, Jonas Sjölund, Magdalena Wolska, Jenny Karlsson, Javanshir Esfandyari, Kristian Pietras, Kristina Aaltonen, David Gisselsson, Daniel Bexell

**Affiliations:** a Division of Translational Cancer Research, Department of Laboratory Medicine, Lund University, Lund, Sweden; b Division of Clinical Genetics, Department of Laboratory Medicine, Lund University, Lund, Sweden; c Department of Pathology, Laboratory Medicine, Medical Services, University Hospital, Lund, Sweden

**Keywords:** Neuroblastoma, Rigosertib, ON-01910, ON-01910.Na, Patient-derived xenografts

## Abstract

High-risk neuroblastoma has a poor prognosis despite intense treatment, demonstrating the need for new therapeutic strategies. Here we evaluated the effects of rigosertib (ON-01910.Na) in preclinical models of high-risk neuroblastoma. Among several hundred cancer cell lines representing 24 tumor types, neuroblastoma was the most sensitive to rigosertib. Treatment of *MYCN*-amplified neuroblastoma organoids resulted in organoid disintegration, decreased cell viability, and increased apoptotic cell death. Neuroblastoma response to rigosertib involved G2M cell cycle arrest and decreased phosphorylation of AKT (Ser473) and ERK1/2 (Thr202/Tyr204). Rigosertib delayed tumor growth and prolonged survival of mice carrying neuroblastoma *MYCN*-amplified PDX tumors (median survival: 31 days, treated; 22 days, vehicle) accompanied with increased apoptosis in treated tumors. We further identified vincristine and rigosertib as a potential promising drug combination treatment. Our results show that rigosertib might be a useful therapeutic agent for *MYCN*-amplified neuroblastomas, especially in combination with existing agents.

## Highlights

• Analysis of multiple tumor types reveal neuroblastoma sensitivity to rigosertib.
• Neuroblastoma 2D and 3D organoid models are sensitive to rigosertib.
• Rigosertib causes G2M cell cycle arrest rather than inhibition of Ras binding domains.
• Rigosertib prolongs survival of *MYCN*- amplified patient-derived xenograft tumors.
• Combination of rigosertib and vincristine is synergistic against neuroblastoma.

## Introduction

Neuroblastoma is responsible for 15% of all pediatric oncology deaths and is the most common solid tumor in children under one year of age. High-risk neuroblastoma is associated with chromosomal alterations including 1p deletion, 11q deletion, 17q gain, *MYCN* amplification, and ALK-activating mutations. High-dose chemotherapy in the rapid COJEC regimen (*i.e.*, cisplatin, etoposide, vincristine, carboplatin, cyclophosphamide), surgery, and other treatment strategies are usually effective against primary disease, but many patients relapse with metastatic disease [1], [2], [3]. Novel therapies for high-risk neuroblastoma are urgently required.

High-risk neuroblastoma contains significant inter- and intratumoral heterogeneity [4], [5], suggesting that multi-signaling inhibition could be a promising and necessary therapeutic strategy. Rigosertib (ON-01910.Na) was originally described as a multi-kinase inhibitor with effects on PLK1 and the phosphoinositide 3-kinase (PI3K) pathway [6], [7], [8], [9]. Rigosertib has also been proposed to function as a RAS mimetic by affecting the RAS-binding domain of RAS effector proteins, thereby inhibiting MEK-ERK signaling [10]. However, recent studies have shown that rigosertib functions as a microtubule-destabilizing agent [11], therefore its precise mechanism(s) of action remain a matter of debate [12], [13]. Nevertheless, rigosertib has shown anti-tumor effects in preclinical *in vivo* models of solid tumors including breast, pancreatic [6], colorectal, and lung cancer [10]. Results from phase 1 clinical studies have reported treatment responses in several tumor types, including ovarian cancer [14], pancreatic ductal adenocarcinoma, thymic carcinoma [15], and head and neck squamous cell carcinoma [16].

In a recent high-throughput drug screen, we identified rigosertib as a potential anti-neuroblastoma agent that displayed high selectivity towards tumor cells compared to healthy bone marrow-derived cells [17]. Recently, Kowalczyk and co-workers also showed that rigosertib can have anti-neuroblastoma effects in conventional *in vitro* neuroblastoma models [18].

We previously established neuroblastoma patient-derived xenograft (PDX) models that retain the geno- and phenotypes of their parental tumors [19], [20]. We also established PDX-derived 3D neuroblastoma organoids that retain the chromosomal aberrations, protein markers, as well as and tumorigenic and metastatic capacities of their parental tumors *in vivo*, making them suitable for preclinical drug testing [19], [21].

Here we used both *in silico* analyses and experimental approaches to investigate neuroblastoma sensitivity to rigosertib. Analysis of drug screening data of hundreds of cancer cell lines from two major public datasets showed that neuroblastoma is one of the most sensitive tumor types to rigosertib. *In vitro*, rigosertib treatment disrupted PDX-derived tumor organoids, decreased cell viability, and induced cell cycle arrest and apoptotic cell death. Transcriptomic analysis (RNA-seq) indicated that rigosertib mainly induces cell cycle arrest. This was accompanied by decreased ERK1/2 (Thr202/Tyr204) and AKT (Ser473) phosphorylation. *In vivo*, rigosertib treatment of a high-risk *MYCN*-amplified PDX model delayed tumor growth and prolonged survival. We also identify drugs which, in combination with rigosertib, might improve its therapeutic efficacy.

## Materials and methods

### Exploration of publicly available drug screening data

The current results are partially based upon data generated by the Cancer Target Discovery and Development (CTD^2^) Network (https://ocg.cancer.gov/programs/ctd2/data-portal) established by the National Cancer Institute's Office of Cancer Genomics. Two publicly available datasets were used: PRISM Repurposing Secondary Screen (BRD-K55187425–236–05–2) and the CTD^2^ dataset (CTRP:660,397). The datasets were accessed through DepMap Portal (https://depmap.org/portal/). Information about the included neuroblastoma cell lines is presented in Table 1. Rigosertib area under the (dose-response) curves (AUCs) of neuroblastoma cell lines were compared with the AUCs of the other cell lines within each dataset.

**Table 1:** Neuroblastoma model characterization. Data acquired from DepMap portal.

**CTD^2^**	***MYCN* status**	**Type**
KELLY	+	Primary
CHP-126	+	Primary
NH-6	+	Metastasis
KP-N-YN	+	Metastasis
MHH-NB-11	+	Metastasis
NB-1	+	Metastasis
SiMa	+	Relapse
SK-N-BE(2)	+	Metastasis
SK-N-FI	–	Metastasis
KP-N-SI9s	–	Metastasis
SK-N-SH	–	Metastasis
**PRISM Repurposing Secondary Screen**	***MYCN* status**	**Type**
KELLY	+	Primary
NB-1	+	Metastasis
SiMa	+	Relapse
SK-N-BE(2)	+	Metastasis
SK-N-AS	–	Metastasis

### Tumor organoid cultures

Neuroblastoma tumor organoids were previously established from PDX mice and cultured as free-floating 3D tumor organoids in serum-free medium with the addition of epidermal growth factor (EGF) and basic fibroblast growth factor (bFGF) as described [19], [21]. Tumor organoids were regularly verified by single nucleotide polymorphism (SNP) analysis and tested for *Mycoplasma*. All *in vitro* experiments were performed in duplicate unless otherwise stated.

### Cell viability assays

LU-NB-1, LU-NB-2, and LU-NB-3 PDX-derived tumor organoids were dissociated into single cells and seeded into opaque 96-well plates (Corning Inc., Corning, NY), 5000 cells per well, and treated immediately with a range (0–100 nM) of rigosertib concentrations. Cells were incubated for 72 h. Cell viability was calculated as a percentage of control wells based on CellTiter-Glo (G7571; Promega, Madison, WI) luminescence. Luminescence was measured with a Synergy2 Multi-Mode plate reader (BioTek, Winooski, VT). Biological triplicates were used.

### Cell death assays

Neuroblastoma PDX cells (1 × 10^6^ cells/ sample) were treated with an ~IC_90_ concentration of rigosertib or control amount of DMSO. Cells were allowed to grow for 72 h, dissociated to single cells using accutase, and stained with cell death markers annexin V and propidium iodide (PI). To investigate the mechanism of cell death, a fluorescent substrate of caspase 3/7 (Nucview 405 1:5, Biotium, Fremont, CA) was used. Flow cytometry was performed with a FACSVerse flow cytometer (BD Biosciences, Franklin Lakes, NJ), and the data were analyzed using the FlowJo software.

### RNA sequencing and analysis

LU-NB-2 PDX cells were seeded in T25 flasks, allowed to grow for 24 h, and treated for 24 h with rigosertib (175 nM, *n* = 6) or DMSO (10 µl, *n* = 6). Cell pellets were then collected and RNA was extracted using the AllPrep DNA/RNA Mini Kit (Qiagen, Hilden, Germany) and sequenced on a NovaSeq 6000 System (20,012,850, Illumina, San Diego, CA). Data is available at R2 (https://hgserver1.amc.nl/cgi-bin/r2/main.cgi) under: ‘PDX Neuroblastoma Rigosertib-in-vitro_LUNB2_2019 - Aaltonen - 12 - custom - ensh38e94’. Differentially expressed genes (ANOVA, *p*<0.01, FDR correction) were subjected to GO term analysis via R2, and obtained significant terms (*p*<0.05) were grouped into higher ranked GO terms groups with QuickGO (https://www.ebi.ac.uk/QuickGO/). Additionally, all differentially expressed genes were subjected to analysis via Gene Set Enrichment Analysis (GSEA) 4.0.3 (Broad Institute) and a collection of Hallmark gene sets from the Molecular Signatures Database was used as a reference. Normalized enrichment scores were used as an indicator of particular pathway enrichment.

### Western blotting

PDX cells were seeded and treated after 24 h with 175 nM of rigosertib for 6, 12, and 24 h. Cells were lysed in RIPA buffer supplemented with complete protease inhibitor (Roche, Basel, Switzerland) and phosSTOP (Roche). Proteins were separated using SDS-PAGE gels and transferred to PVDF or Hybond‐C extra nitrocellulose membranes (Bio-Rad Laboratories, Hercules, CA). Antibodies used: pAkt (Ser473) (#4060S, Cell Signaling Technology, Danvers, MA; 1:2000), panAkt (#2920S, Cell Signaling Technology; 1:2000), pErk1/2 (Thr202/Tyr204) (#4377S, Cell Signaling Technology; 1:1000), total Erk1/2 (#4696, Cell Signaling Technology, 1:1000), c-Raf (Ser338 phosphorylated) (#9427, Cell Signaling Technology; 1:1000), total c-Raf (#610,151, BD BioSciences, San Jose, CA; 1:1000), MYCN (#Ab24193, Abcam, Cambridge, UK; 1:1000), and Actin (#691001, MP Biomedicals, Santa Ana, CA; 1:2000) or SDHA (#Ab14715, Abcam; 1:4000) as loading controls. Phosphorylated and total proteins were probed on the same membrane. In the case of Akt and c-Raf, differences in secondary antibodies (mouse and rabbit) were utilized. Before the total Erk read, pErk antibody was stripped using Restore Western Blot Stripping Buffer (Thermo Scientific, #21059) with protocol recommended by the manufacturer. All membranes were imaged using Luminata Forte Western HRP substrate (Merck Millipore, #10394675 and Amersham Imager 600 (GE Healthcare Bio-Sciences AB).

### Combination drug testing

Tumor organoids were dissociated into single cells and seeded into 96-well plates (20 000 cells per well in a volume of 80 μl). The cells were allowed to form organoids for 48 h and were then treated with 10 μl of rigosertib and 10 μl of the additional drug (cisplatin, vincristine, filanesib, or azacitidine) at varying concentrations (see matrices). The tumor organoids were incubated for 72 h and then analyzed for cell death (released proteases) and cell viability using the CytoTox-Glo (G9290; Promega, Madison, WI) assay according to the manufacturer's instructions. Cell viability matrices were used to calculate most synergistic area scores (MSA) using FIMM SynergyFinder (https://synergyfinder.fimm.fi) [22]. SynergyFinder compares the observed drug combination responses to the expected responses calculated using a synergy modeling method (here ZIP scores) [23]. The predicted response is compared with experimental data and the synergy score (δ) is calculated as percent of response beyond expectation.

### *In vivo* study

*In vivo* studies were conducted according to the guidelines from the regional Ethics Committee for Animal Research in Lund/Malmö (ethical permit no. M11–15). LU-NB-3 PDX tumor cells (2 × 10^6^) were suspended in medium/Matrigel (2:1) and injected subcutaneously into the flanks of female nude mice. Each mouse was allocated to either the control or treated group when the tumor reached 200–300 mm^3^. Tumor volume in mm^3^ was measured with a caliper and calculated according to the formula $V=\frac{\pi \left(length\right){\left(width\right)}^{2}}{6}.$ Mice were treated intraperitoneally (i.p.) five times a week with PBS (*n* = 7) or PBS-diluted rigosertib: 200 mg/kg (*n* = 7). When tumors exceeded 1800 mm^3^, mice were sacrificed and tumor pieces were collected, fixed in 4% paraformaldehyde, and embedded in paraffin.

### Immunohistochemistry

PDX tumor sections (4 μm) were stained manually with hematoxylin and eosin (H&E). H&E staining of all tumor sections was assessed by blinded analysis. Tumor sections (*n* = 5, control; *n* = 7, treated) were analyzed for cell death using the TUNEL Assay Kit HRP-DAB (Abcam ab206386). Three representative photos of each slide were subjected to CellProfiler 3.1.8 analysis, and the numbers of DAB positive cells were quantified [24].

### Statistical analysis

Statistical analysis of the data was performed with GraphPad Prism version 8.0.1 for Windows (GraphPad Software, San Diego, CA) or Excel 2016 (Microsoft). The two-sided unpaired *t*-test was used to analyze differences between groups. Statistical significance for Kaplan-Meier survival analyses was calculated using the log-rank test. Data from RNA sequencing was analyzed using one-way analysis of variance (ANOVA) with a 0.01 false discovery rate (FDR) correction for multiple testing.

## Results

### Multiple neuroblastoma models show high sensitivity to rigosertib

**Fig. 1:**
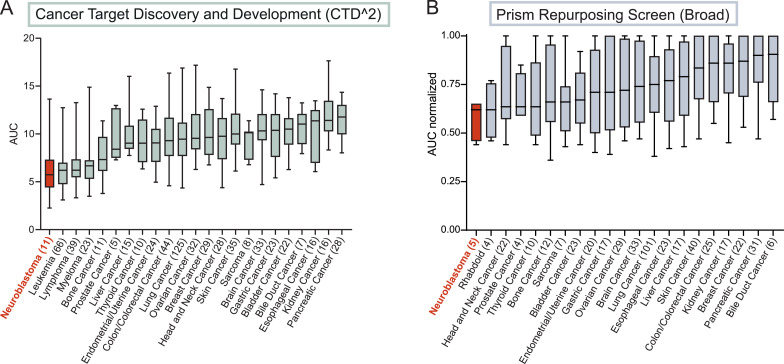
Rigosertib sensitivity across different cancer types. **A)** Sensitivity to rigosertib obtained from dose-response data across 650 tumor cell lines representing 23 tumor types. Data were obtained from the Cancer Target Discovery and Development (CTD^2^) network. Box plots show mean rigosertib sensitivity values. Whiskers represent min and max values; each box represents the interquartile range. The numbers in parentheses represent the numbers of cell lines for each tumor type. Values were obtained from the DepMap portal. AUC, area under the curve. **B)** Sensitivity to rigosertib obtained from dose-response data across 468 tumor cell lines representing 21 tumor types. Data obtained from the PRISM Repurposing Screen at the Broad Institute. Box plots show mean rigosertib sensitivity values. Whiskers represent min and max values; the box represents the interquartile range. The numbers in parentheses represent the numbers of cell lines for each tumor type. Values were obtained from the DepMap portal, where they were normalized to concentration range. AUC normalized, normalized area under the curve.

We analyzed rigosertib sensitivity in a wide range of cancer types using publicly available screening data: Cancer Target Discovery and Development (CTD^2^, CTPR 660397) [25], [26], containing 650 tumor cell lines representing 23 tumor types, and PRISM Repurposing Primary Screen 19Q4 [27], containing 468 tumor cell lines representing 21 tumor types (https://depmap.org/portal/compound/rigosertib?tab=overview). These datasets include *MYCN*-amplified and non-*MYCN* amplified neuroblastoma models representing one relapse, two primary tumors, and 10 metastases (Table 1). Neuroblastoma had the lowest mean Area Under the Curve (AUC), thus making it the most rigosertib-sensitive cancer type across all tumor types in both datasets (Fig. 1**A-B**). There was no obvious difference of IC50 values between *MYCN*-amplified and non-*MYCN* amplified cell lines in the CTD^2^ dataset (**Fig. S1**). In a recent drug screen, we assessed drug sensitivity scores for various compounds against three neuroblastoma PDX models (LU-NB-1, LU-NB-2, and LU-NB-3) relative to human healthy bone marrow cells [17]. There was a more than three-fold increase in the effect of rigosertib on neuroblastoma cells relative to the effect on bone marrow control cells (**Fig. S2**). Rigosertib is thus effective in a wide range of preclinical high-risk neuroblastoma models, with low effect on healthy bone marrow cells, suggesting an underlying vulnerability to rigosertib in neuroblastomas (https://depmap.org/portal/compound/rigosertib?tab=overview).

### Rigosertib treatment of neuroblastoma organoids results in their disintegration and apoptosis

**Fig. 2:**
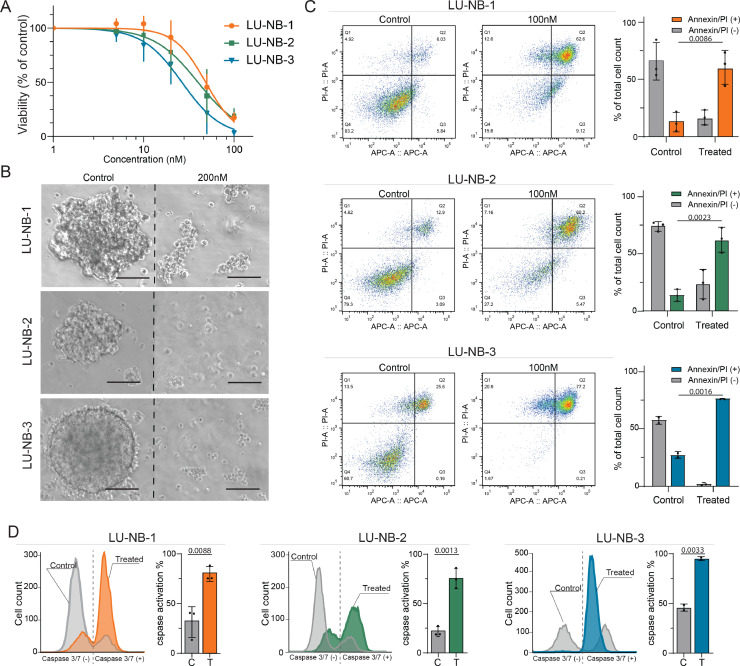
Rigosertib treatment of neuroblastoma organoids. **A)** Dose-response curves of neuroblastoma PDX cells LU-NB-1, LU-NB-2, and LU-NB-3 after rigosertib treatment (72 h). Data presented as percentage of tumor cell viability relative to DMSO control; mean ± SD of three independent experiments. **B)** Morphology of neuroblastoma organoids following rigosertib treatment (200 nM, 72 h). **C)** Annexin and PI flow cytometry of neuroblastoma PDX cells treated with rigosertib (100 nM, 72 h). Quantitative analysis of live cells and PI-positive cells. Bars show the mean ± SD of two or three replicates. **D)** Analysis of caspase-3/7 substrate activation following rigosertib treatment (100 nM, 72 h) and quantitative analysis of apoptotic cells. Bars show the mean ± SD of two or three replicates. Scale bar = 100 µM. C, control; T, treated.

We next tested rigosertib in PDX-derived neuroblastoma organoids. These tumor organoids originate from high-risk tumors containing 1p loss, *MYCN* amplification, and 17q gain and are tumorigenic upon implantation *in vivo* (Table 2) [19], [21]. The average rigosertib IC_50_s for the three PDX neuroblastoma organoids LU-NB-1, LU-NB-2, and LU-NB-3 based on tumor cell viability were 48.5 nM, 39.9 nM, and 26.5 nM, respectively (Fig. 2**A**). Rigosertib treatment of neuroblastoma organoids required a higher drug concentration (200 nM for 72 h) and resulted in complete disintegration of their 3D structures (Fig. 2**B**). Furthermore, treatment (100 nM for 72 h) caused an increased fraction of late apoptotic/necrotic cells (annexin V and PI-positive cells) compared to control DMSO treated sample (Fig. 2**C**). To examine the possible mechanism of cell death triggered by rigosertib, we performed flow cytometry with fluorescent caspase-3/7 substrate and observed higher levels of reagent activation in the treated samples, indicating apoptosis as an active cell death pathway (Fig. 2**D**).

**Table 2:** Patient tumor characteristics. UD = undifferentiated, PD= poorly differentiated.

**Tumor**	**Age**	**Type**	**Chemotherapy**	**INSS stage**	**Histology**	**SNP profile**
LU-NB-1	1y4m	Primary	No	IV	UD	*MYCN* amp, 1p-, +17q
LU-NB-2	2y2m	Metastasis	Yes	IV	UD	*MYCN* amp, 1p-, +17q
LU-NB-3	2y9m	Primary	No	III	PD	*MYCN* amp, 1p-, +17q

### Neuroblastoma response to rigosertib treatment

**Fig. 3:**
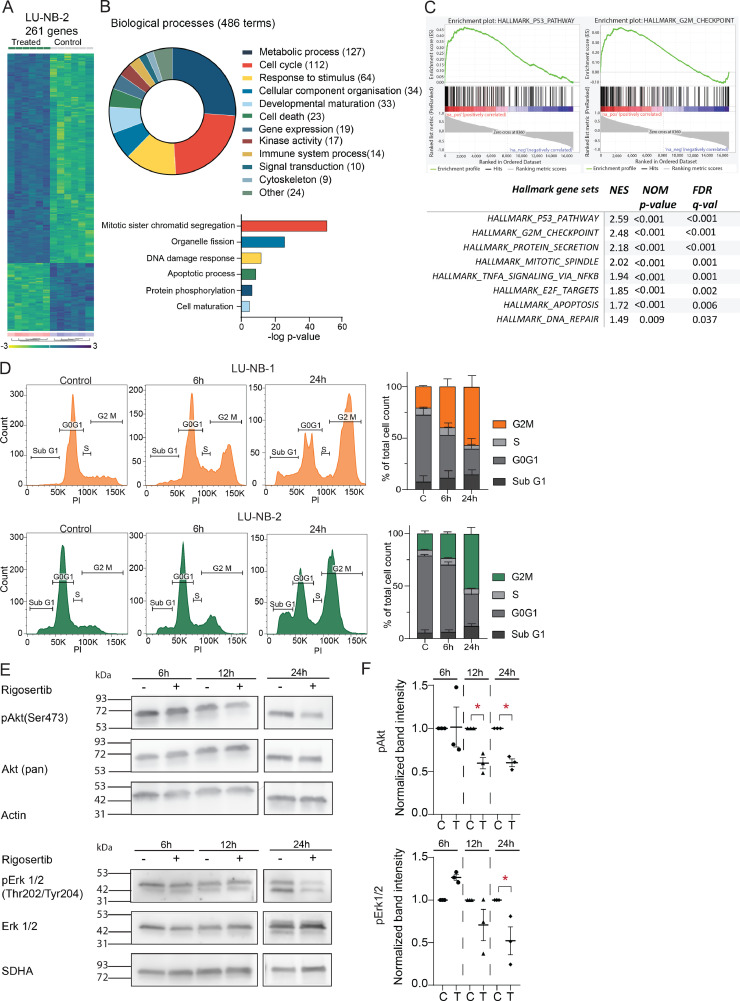
Neuroblastoma response to rigosertib treatment. **A)** Supervised transcriptomic analysis of treated cells (175 nM rigosertib for 24 h) and controls (*n* = 6 for each group; ANOVA, FDR correction, *p*<0.01). 261 genes were differentially expressed (199 genes upregulated and 62 genes downregulated). **B)** Gene Ontology (GO) analysis of the 261 differentially expressed genes. The pie chart shows the proportions of biological processes (*p* < 0.05). The most significant GO terms for each group are shown below with respective p-values. **C)** Gene Set Enrichment Analysis (GSEA) of all differentially expressed genes showing the highest relevant enrichment scores from the Hallmark dataset. **D)** Flow cytometry cell cycle analysis of LU-NB-1 and LU-NB-2 PDX cells. Cells were treated with rigosertib (175 nM) and analyzed by flow cytometry at 6 h and 24 h. Representative histograms are followed by summary of cell cycle distribution of three replicates with SD. **E)** Western blot analysis of pAkt (Ser473) and pErk1/2 (Thr202/Tyr204). LU-NB-1 PDX cells were treated with rigosertib (175 nM) and analyzed at 6 h, 12 h, and 24 h. **F)** Quantitative analysis of Western blots shown in (E). Samples were normalized to the total amount of protein and loading controls. Statistical analysis was performed using two-sided unpaired *t*-test, *p<0.05.

To elucidate the transcriptional response of rigosertib in neuroblastoma, we performed RNA-seq of LU-NB-2 PDX cells treated with rigosertib (175 nM) for 24 h (*n* = 6 treated, *n* = 6 controls). Supervised clustering of the two groups revealed 261 differentially expressed genes (199 upregulated, 62 downregulated; ANOVA *p*<0.01 with FDR correction; Fig. 3**A**). Gene ontology (GO) analysis revealed engagement of multiple biological processes during rigosertib treatment including metabolic processes (127 terms), cell cycle (112 terms), response to stimulus (64 terms), cellular component organization (34 terms) , developmental maturation (33 terms), and cell death (23 terms) (Fig. 3**B**). The most significant specific processes in each group were protein phosphorylation (*p* = 3.8^e^^-^^7^), mitotic sister chromatid segregation (*p* = 5.1^e-52^), DNA damage response (signal transduction by p53 class mediator; *p* = 1.9^e-12^), organelle fission (*p* = 2.7^e-26^), cell maturation (*p* = 1.5^e-5^), and apoptotic process (*p* = 2.5^e-9^; chi-square test, continuity correction; Fig. 3**B**). Gene Set Enrichment Analysis (GSEA) showed that processes involved in neuroblastoma response to rigosertib included the *TP53* pathway, G2M checkpoint, mitotic spindle, and TNF-α signaling via NF-κB (Fig. 3**C**).

Flow cytometric cell cycle analysis showed that rigosertib treatment of LU-NB-1 and LU-NB-2 neuroblastoma cells increased the fraction of cells in G2/M phase (Fig. 3**D**), indicating inhibition of mitosis. There was also an increase of cells in the sub-G1 phase, representing dead cells, particularly at 24 h (Fig. 3**D**).

We performed Western blot analysis to further investigate specific rigosertib-induced pathways. LU-NB-1 PDX cells were treated with rigosertib (175 nM) and analyzed at 6, 12, and 24 h. pAKT (Ser473) and pERK1/2 (Thr202/Tyr204) protein levels decreased at 24 h (Fig. 3**E**). However, c-RAF (Ser338 phosphorylated) levels and N-Myc protein levels were unchanged at 24 h (**Figs. S3A, S3B**).

### Rigosertib has anti-neuroblastoma effects in a PDX model *in vivo*

**Fig. 4:**
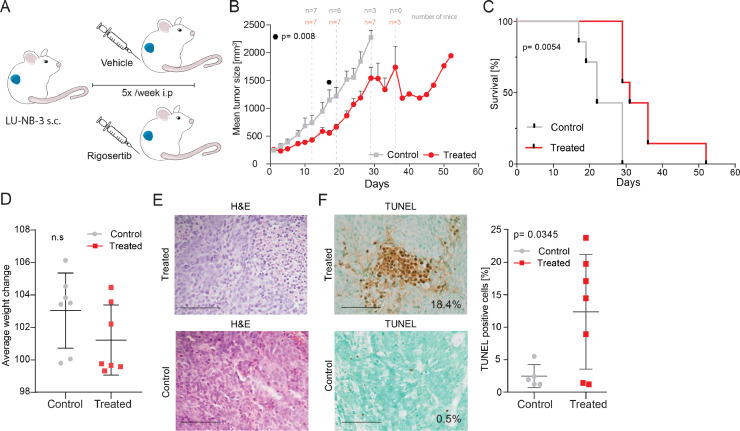
Rigosertib treatment of a high-risk neuroblastoma PDX model *in vivo*. **A)** LU-NB-3 PDX cells were injected subcutaneously into nude mice. Mice were allocated to treatment or vehicle groups when the tumor reached at least 200 mm^3^. Mice were treated with rigosertib (200 mg/kg i.p.) or vehicle five times per week until they were sacrificed. **B)** Mean tumor size of the vehicle group (*n* = 7) and treated group (*n* = 7). The symbol shows significant difference at day 17 (*p* = 0.008), two-sided unpaired *t*-test. The numbers of mice at different time points are presented. **C)** Kaplan-Meier graph showing the survival of treated mice and controls. Median survival was 31 days for treated mice and 22 days for controls. **D)** Average mouse weight during treatment shown as percent of initial weight. **E)** H&E staining of representative tumor sections from treated mice and controls. **F)** Cell death analysis (TUNEL) of representative tumor sections from treated mice and controls. Each data point represents a mean of three representative x40 views. Statistical analysis was performed using the two-sided unpaired *t*-test. Scale bar = 100 µM.

We treated tumor-carrying nude mice with rigosertib (intraperitoneally (i.p.) 200 mg/kg). LU-NB-3 PDX tumors were established subcutaneously by injecting 2 × 10^6^ cells. Mice were individually allocated to treatment or control (vehicle) groups when tumors reached at least 200 mm^3^ (mean tumor size 254 mm^3^). Mice were treated with either rigosertib (200 mg/kg i.p.) or with ~110 µl PBS solution (vehicle) five times per week (Fig. 4**A**). Rigosertib significantly delayed tumor growth compared to vehicle (*p* = 0.008 at day 17; Figs. 4**B, S4A**). Furthermore, rigosertib-treated mice lived significantly longer than vehicle treated (*p* = 0.0054; median survival: 31 days, rigosertib treated; 22 days, vehicle treated; Fig. 4**C**). All mice tolerated treatment without significant weight loss as compared to the control mice (Figs. 4**D, S4B**). Analysis of H&E-stained tumor sections showed that both groups contained small round blue tumor cells with undifferentiated morphology. (Fig. 4**E**). TUNEL staining of tumor sections revealed an increased number of dead cells in the treated tumors compared to vehicle (*p* = 0.035, Figs. 4**F, S4C**). Thus, rigosertib treatment can (i) delay neuroblastoma PDX growth *in vivo*; (ii) increase survival of neuroblastoma-carrying mice, and (iii) increase TUNEL-positive apoptotic cells *in vivo*.

### Combination drug testing

**Fig. 5:**
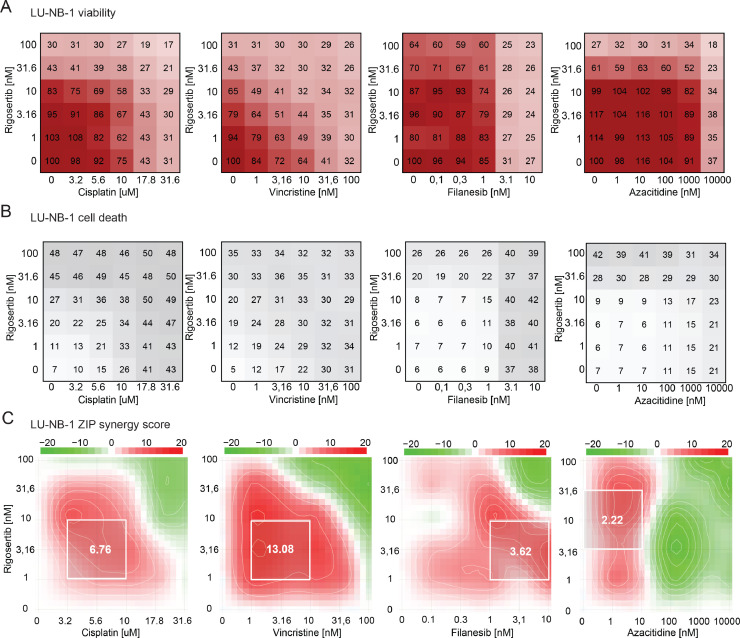
Drug combination testing indicates new treatment combinations. **A)** Rigosertib in combination with cisplatin, vincristine, filanesib, and azacitidine were tested on LU-NB-1 tumor organoids using 6 × 6 matrices of different drug concentrations and assessed for tumor cell viability. **B)** Drugs and concentrations as in (A) analyzed for tumor cell death. **C)** Drug synergy calculations (using the ZIP synergy model) based on cell viability matrices in (A). The white square represents most synergistic area score.

We next examined potential drug combinations to enhance efficacy. Rigosertib was combined with cisplatin or vincristine (included in the clinically used rapid-COJEC regimen), filanesib (a mitotic inhibitor with strong anti-neuroblastoma effects [17]), or azacitidine (effective in combination with rigosertib in other malignancies [28]). Combination testing was performed in LU-NB-1 and LU-NB-2 models using 6 × 6 matrices and cell viability and cell death analyses (Figs. 5**A, B, S5A, B**). ZIP synergy scores were calculated based on cell viability (Figs. 5**C, S5C**). The most synergistic area (MSA) for each drug combination was, for LU-NB-1, 6.76 (cisplatin), 13.08 (vincristine), 3.62 (filanesib), and 2.22 (azacitidine) **(**Fig. 5**A)**; and for LU-NB-2, 2.71 (cisplatin), 4.56 (vincristine), 3.54 (filanesib), and 6.89 (azacitidine) **(Fig. S5C)**. The results indicate synergistic or additive effects of the drug combinations. In particular, rigosertib in combination with vincristine might be a beneficial drug combination strategy.

## Discussion

In a recent high-throughput drug screen, we identified rigosertib as one of the top tumor-selective drugs against neuroblastoma [17]. In the present study, we analyzed data from hundreds of cancer cell lines covering multiple tumor types and identified neuroblastoma as a tumor type sensitive to rigosertib. Treatment of PDX-derived neuroblastoma organoids disrupted the 3D structure, decreased cell viability, increased cell death, induced cell cycle arrest, and downregulated mediators of the RAS and AKT signaling pathway. In a *MYCN*-amplified neuroblastoma PDX model, rigosertib delayed tumor growth and prolonged mouse survival. We also identified rigosertib and vincristine as a potential drug combination. The mild side-effects of rigosertib in patients [16], [29], its availability as oral formulations, and the results presented here suggest that rigosertib could form part of novel anti-neuroblastoma drug combination strategies.

Initial findings described rigosertib as a multi-kinase signaling inhibitor with effects on PLK1, PI3K pathways, and the RAF-MEK-ERK pathway [7], [30], [31], [32], but the precise mechanism of action of rigosertib is still being debated [11], [12], [13]. Our results showing decreased pAKT (Ser473) and pERK1/2 (Thr202/Tyr204) levels indicate the involvement of the AKT and RAS signaling pathways in its mechanism of action. However, our RNA-seq results are consistent with recent CRISPRi/a-based chemical genetic screens and targeted cell biological, biochemical, and structural assays [11], [13] suggesting that rigosertib functions as a microtubule-destabilizing agent. Our finding that c-RAF (Ser338 phosphorylated) levels were unchanged by rigosertib might indicate that the decreases in pAKT (Ser473) and pERK1/2 (Thr202/204) were not caused by upstream blockage of RAS effectors but rather indirect effects due to other mechanisms like microtubule inhibition and cell cycle arrest. This is consistent with recent findings suggesting that rigosertib has tubulin-interacting effects rather than RAS signaling inhibitory effects in neuroblastoma [18]. It has also recently been reported that several cancer drugs in clinical trials do not function as previously thought but rather through off target-effects [33], and this cannot be ruled out for rigosertib. Clarification of the precise mechanism of action will help to design rational therapeutic combination strategies and companion biomarkers for patient identification and selection.

Using a *MYCN*-amplified neuroblastoma PDX model, we observed that rigosertib can delay tumor growth *in vivo*, accompanied by an increase in apoptotic cells. This is in contrast to a recent study by Kowalczyk et al., in which rigosertib had no *in vivo* effects when using the conventional cell line SK-N-AS [18]. This discrepancy might reflect the fact that neuroblastoma is very heterogenous and drug responses can vary between patients. As shown in preclinical and clinical studies, rigosertib is quickly eliminated from plasma (t1/2 ~30 min in mice, ~2 h in humans) which might contribute to its modest response as a single drug [34], [35]. Development of extended release formulations could increase tumor exposure of the drug and improve anti-tumor efficacy. Our findings point to further need for therapy optimization as complete regression is rarely obtained with single agent treatment. We investigated clinically relevant drugs in combination with rigosertib and identified synergistic effects between rigosertib and vincristine, an antimitotic agent included in the rapid-COJEC regimen.

Taken together, we show that rigosertib has anti-neuroblastoma effects in multiple preclinical models. Rigosertib decreased neuroblastoma cell viability and caused cell cycle arrest and cell death through apoptosis. *In vivo,* treatment was well-tolerated, delayed tumor growth and increased survival of mice. Additionally, combination therapy with vincristine might improve responses and should be further investigated. The mode of action of rigosertib is still being debated and needs to be resolved. Nevertheless, our findings suggest that rigosertib may add benefit as part of a combination treatment for *MYCN*-amplified high-risk neuroblastomas.

## CRediT authorship contribution statement

**Katarzyna Radke:** Conceptualization, Formal analysis, Investigation, Writing – original draft, Visualization. **Karin Hansson:** Conceptualization, Investigation. **Jonas Sjölund:** Formal analysis, Investigation, Data curation. **Magdalena Wolska:** Investigation. **Jenny Karlsson:** Investigation, Data curation. **Javanshir Esfandyari:** Investigation. **Kristian Pietras:** Formal analysis. **Kristina Aaltonen:** Formal analysis, Data curation. **David Gisselsson:** Formal analysis. **Daniel Bexell:** Conceptualization, Writing – original draft, Funding acquisition, Supervision.

## Declaration of Competing Interest

The authors declare no conflicts of interest.

## Appendix Supplementary materials

Image, application 1
