# Supplementary material for: Anti-tumor effects of rigosertib in high-risk neuroblastoma
Source: Transl Oncol. 2021 Jun 9;14(8):101149. doi: 10.1016/j.tranon.2021.101149 (PMC8207190; doi:10.1016/j.tranon.2021.101149)
Supplement: Supplementary file 1 [file mmc1.docx]

**Radke et al, Supplemental figures**

**Figure S1**. **Effects of rigosertib on neuroblastoma cell lines.**

Publicly available data showing dose-response curves of rigosertib treatment of non-MYCN-amplified (red) and MYCN-amplified (black) neuroblastoma cell lines. Each data point is represented by two replicates. Data obtained from CTD^2^.


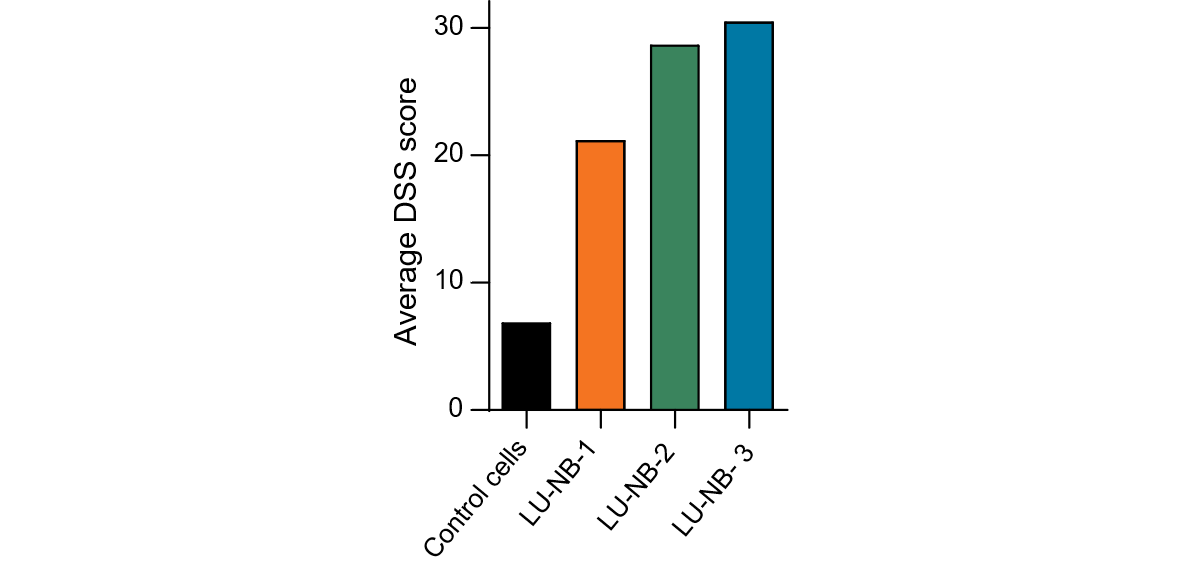


**Figure S2.** **Rigosertib treatment on healthy cells relative to neuroblastoma cells.**

Average drug sensitivity score (DSS) for human healthy bone marrow-derived mononuclear cells (control cells) as well as neuroblastoma PDX-derived tumor organoids LU-NB-1, LU-NB-2 and LU-NB-3. Data is obtained from Hansson et al., *Science Transl Med*, 2020.


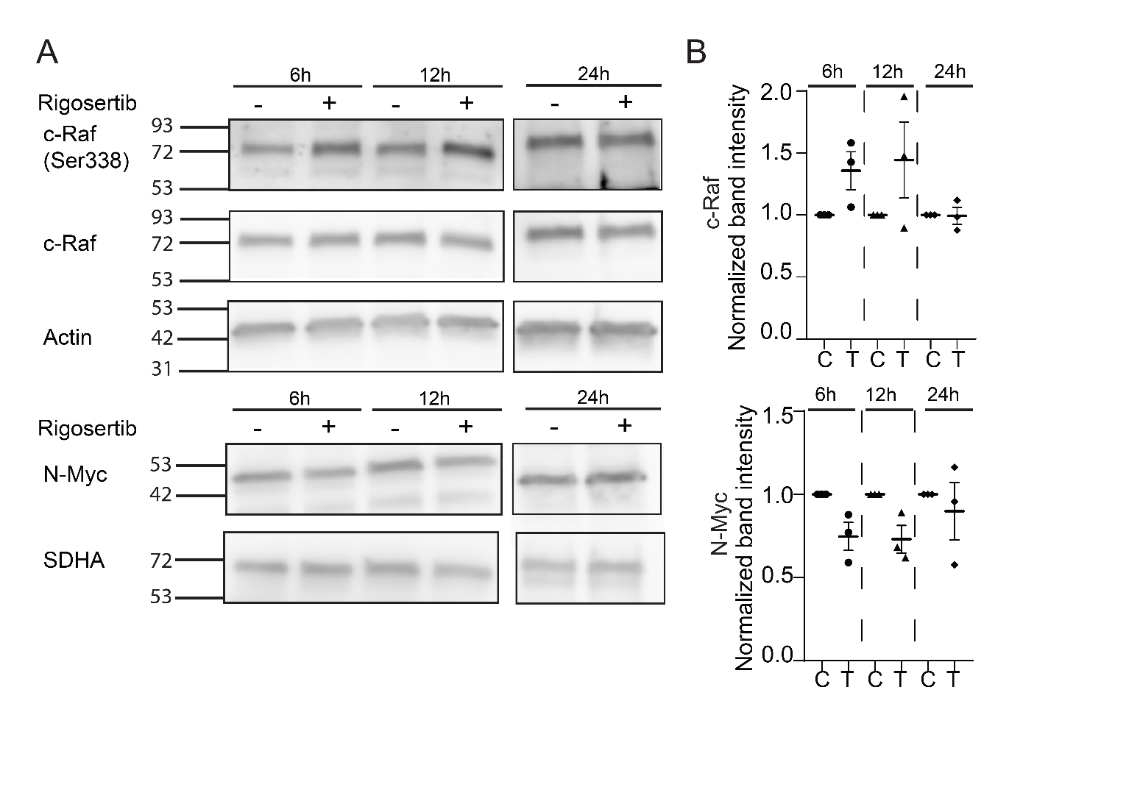


**Figure S3. Analysis of pcRAF and N-MYC protein levels following rigosertib treatment.**

**A)** Western blot analysis of c-RAF (Ser338 phosphorylated) and N-Myc levels. LU-NB-1 PDX cells were treated with rigosertib (175 nM) and analyzed at 6 h, 12 h, and 24 h. **B)** Quantitative analysis of western blots shown in (A). Samples were normalized to the total amount of protein and loading controls.

**Figure S4. Individual response to rigosertib treatment *in vivo*.**

**A)** Tumor size of each individual mouse during rigosertib treatment (200 mg/kg i.p.) *in vivo*. **B)** Weight of each individual mouse during rigosertib treatment presented as a ratio to initial weight. **C)** Percentage of TUNEL-positive cells in vehicle-treated tumors (V1-V6) and in rigosertib-treated tumors (T1-T7). Three slides per tumor were analyzed.


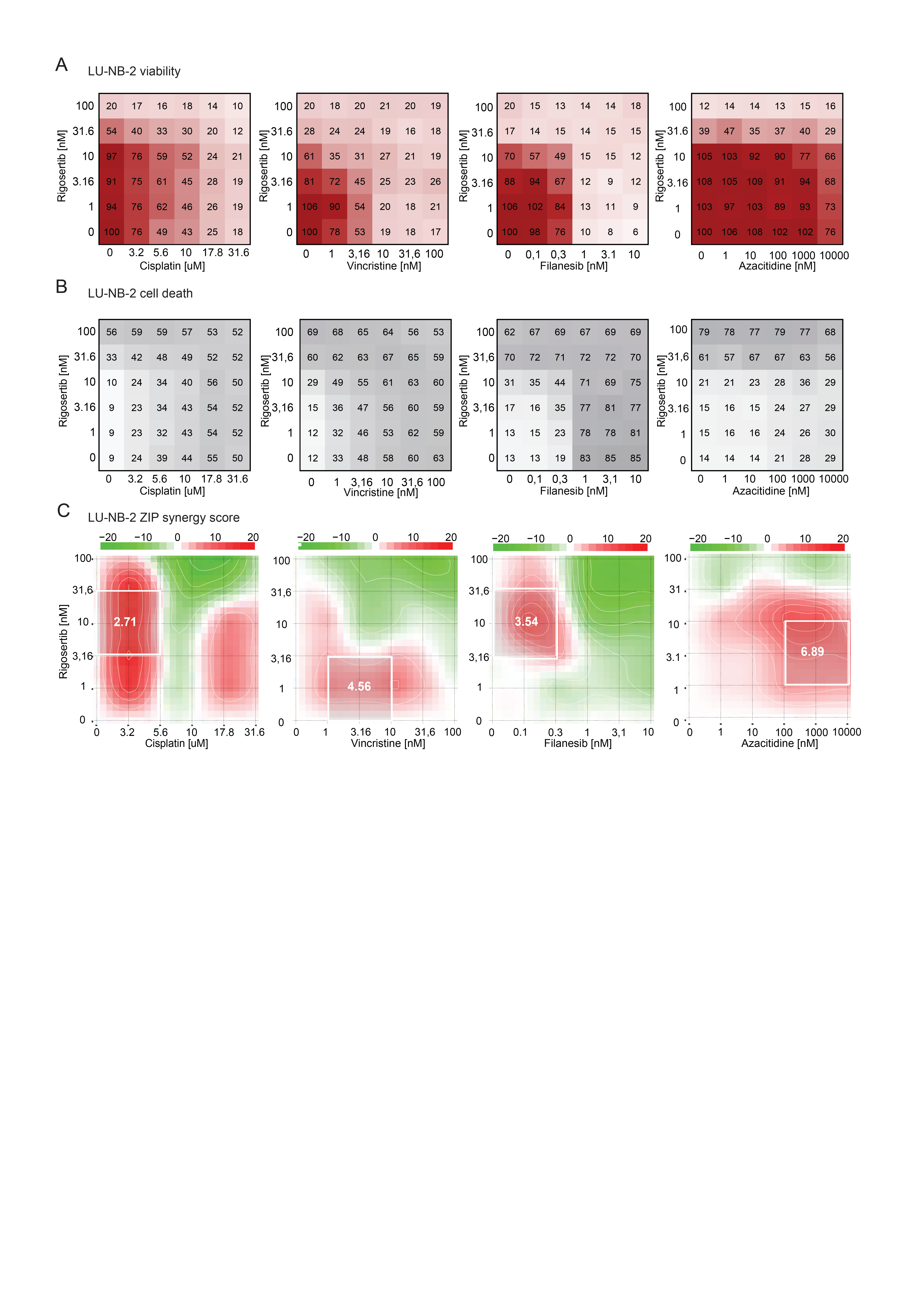


**Figure S5. LU-NB-2 drug combination testing indicates new treatment combinations.**

**A)** Rigosertib in combination with cisplatin, vincristine, filanesib and azacitidine were tested on LU-NB-2 tumor organoids using 6 x 6 matrices of different drug concentrations and assessed for tumor cell viability. **B)** Drugs and concentrations as in (A) analyzed for tumor cell death. **C)** Drug synergy calculations (using the ZIP synergy model) based on cell viability matrices in (A). The white square represents most synergistic area score.
